# A simplified primary aldosteronism surgical outcome score is a useful prediction model when target organ damage is unknown – Retrospective cohort study

**DOI:** 10.1016/j.amsu.2021.102333

**Published:** 2021-04-20

**Authors:** Diederik P.D. Suurd, Wouter P. Visscher, Wessel M.C.M. Vorselaars, Dirk-Jan van Beek, Wilko Spiering, Inne H.M. Borel Rinkes, Gerlof D. Valk, Menno R. Vriens, International CONNsortium Study Group, Rasa Zarnegar, Thomas J. Fahey, Quan Y. Duh, Wen T. Shen, Frederick T. Drake, David B. McAneny, Catherine McManus, James A. Lee, Scott B. Grant, Raymon H. Grogan, Minerva A. Romero Arenas, Nancy D. Perrier, Cord Sturgeon, Tanya Castelino, Elliot J. Mitmaker, David N. Parente, Jesse D. Pasternak, Stan B. Sidhu, Mark Sywak, Gerardo D'Amato, Marco Raffaelli, Valerie Schuermans, Nicole D. Bouvy, Hasan H. Eker, H. Jaap Bonjer, Anton F. Engelsman, Els J.M. Nieveen van Dijkum, Michiel N. Kerstens, Schelto Kruijff

**Affiliations:** d Department of Endocrine and Minimally Invasive Surgery, Weill Cornell Medical College, New York, USA; e Department of Surgery, University of California San Francisco, San Francisco, USA; f Department of Surgery, Boston University School of Medicine and Department of Graduate Medical Sciences, Boston, USA; g Department of Endocrine Surgery, New York-Presbyterian-Columbia University, New York, USA; h Department of Surgery, University of Chicago Medical Center, Chicago, USA; i Department of Endocrine Surgery, Baylor St. Luke's Medical Center, Houston, USA; j Department of Surgical Oncology, University of Texas MD Anderson Cancer Center, Houston, USA; k Department of Surgery, Northwestern University Feinberg School of Medicine, Chicago, USA; l Steinberg-Bernstein Centre for Minimally Invasive Surgery and Innovation, McGill University Health Centre, Montreal, Canada; m Department of Surgery, University Health Network-Toronto General Hospital, Toronto, Canada; n Department of Endocrine Surgery, Royal North Shore Hospital, Sydney, Australia; o Department of Endocrine and Metabolic Surgery, Mater Olbia Hospital, Olbia, Italy; p U.O.C. Chirurgia Endocrina e Metabolica, Fondazione Policlinico Universitario A, Gemelli IRCCS, Rome, Italy; q Instituto di Semeiotica Chirurgica, Facolta di Medicina e Chirurgia, Universita Cattolica del Sacro Cuore, Rome, Italy; r Department of Surgery, Maastricht University Medical Center+, Maastricht, the Netherlands; s Department of Surgery, Amsterdam Gastroenterology and Metabolism, Amsterdam UMC, University of Amsterdam, Amsterdam, the Netherlands; t Department of Endocrinology, University of Groningen, University Medical Center Groningen, Groningen, the Netherlands; u Department of Surgery, University of Groningen, University Medical Center Groningen, Groningen, the Netherlands; a Department of Surgical Oncology and Endocrine Surgery, University Medical Center Utrecht, Heidelberglaan 100, 3584 CX, Utrecht, the Netherlands; b Department of Vascular Medicine, University Medical Center Utrecht, Heidelberglaan 100, 3584 CX, Utrecht, the Netherlands; c Department of Endocrine Oncology, University Medical Center Utrecht, Heidelberglaan 100, 3584 CX, Utrecht, the Netherlands

**Keywords:** Adrenalectomy, Hypertension, Primary aldosteronism, Blood pressure, Endocrine surgery, PASO score

## Abstract

**Background:**

Cure of hypertension after adrenalectomy for primary aldosteronism is no certainty and therefore preoperative patient counseling is essential. The Primary Aldosteronism Surgical Outcome (PASO) Score is a useful prediction model with an area under the curve (AUC) of 0.839. The PASO Score includes ‘Target Organ Damage’ (TOD) (i.e., left ventricular hypertrophy and/or microalbuminuria), which is often unavailable during preoperative counseling and might therefore limit its use in clinical practice. We hypothesized that the PASO score would still be useful if TOD is unknown at time of counseling. Therefore, we aimed to examine the predictive performance of the simplified PASO Score, without taking TOD into account.

**Materials and methods:**

In this retrospective cohort study, patients who underwent unilateral adrenalectomy between 2010 and 2016 in 16 medical centers from North America, Europe and Australia were included. TOD was unknown in our database and therefore assigned as absent. Patients were classified as complete, partial or absent clinical success using the PASO consensus criteria.

**Results:**

A total of 380 (73.9%) patients were eligible for analysis. Complete, partial and absent clinical success were observed in 29.5%, 55.8% and 14.7% of patients, respectively. The simplified PASO Score had an AUC of 0.730 (95% confidence interval 0.674–0.785) in our total cohort.

**Conclusion:**

Without taking TOD into account, the simplified PASO Score had a lower predictive value as compared to the original derivation cohort. Ideally, the complete PASO Score should be used, but when data on TOD are not readily available, the simplified PASO Score is a useful and reasonable alternative.

## Highlights

• We aimed to examine the predictive performance of the PASO Score, without taking ‘target organ damage’ (TOD) into account.
• This simplified PASO Score had a lower predictive value as compared to the PASO Score in the original derivation cohort.
• The simplified PASO Score increases the applicability of the model and is reasonable for clinicians to use in daily practice.
• Ideally, the complete PASO Score should be used, but the simplified PASO Score is a useful and reasonable alternative.

## 1 Introduction

Primary aldosteronism (PA) is the most common form of secondary hypertension. It is estimated that the prevalence of PA in the general hypertensive population is about 5% and up to 20% in more resistant cases of hypertension [[1], [2], [3], [4]]. Hypertension in PA is caused by an excess endogenous production of aldosterone, either due to bilateral adrenal hyperplasia or a unilateral aldosterone producing adenoma (APA) [5,6]. Bilateral adrenal hyperplasia is treated medically with mineralocorticoid receptor antagonist and APA is ideally treated surgically with unilateral adrenalectomy [7,8]. The ultimate goal of adrenalectomy is to resolve excessive aldosterone production, and thereby to cure hypertension allowing the discontinuation of antihypertensive medications [9].

In order to improve preoperative counseling of patients suffering from PA regarding the chance of curation of hypertension after unilateral adrenalectomy, Burrello et al. developed the Primary Aldosteronism Surgical Outcome (PASO) Score [10]. Selecting those patients at highest chance of curation is important because previous studies showed cure rates of only 27%–37% within large, international and well-executed studies [9,11,12]. The PASO Score consists of 6 variables: duration of hypertension, sex, body mass index (BMI), defined daily dose (DDD) of antihypertensive medications, target organ damage (TOD) and largest adrenal nodule at imaging. Each variable is divided in categories which are assigned a varying number of prediction points with a maximum total of 25 points (Table 1). Based on the total score the patients are classified as more likely to achieve complete clinical success (PASO Score > 16) or partial/absent clinical success (PASO Score ≤ 16). In contrast to the Aldosteronoma Resolution Score (ARS), the PASO Score uses DDD instead of number of antihypertensive medications and additionally it includes size of largest adrenal nodule at and TOD besides BMI, duration of hypertension and sex, which are included in both prediction scores [13]. Specifically by including DDD, largest nodule size at imaging and TOD, indicated by micro-albuminuria and/or left ventricle hypertrophy, the investigators achieved an area under the curve (AUC) of 0.839 and an accuracy of 79.2% [9]. However, adequate analysis of the presence of TOD requires electrocardiogram and/or echocardiography to rule out left ventricle hypertrophy and urine assessment to rule out micro-albuminuria. In daily clinical practice these measurements are not routinely performed during preoperative work-up. Consequently, clinicians may have insufficient data to use the PASO Score, which limits its use during patient counseling. In our multicenter real-life cohort TOD was not collected and, probably, not available in most of the patients. This is also the case within the original PASO cohort, since the TOD status was described as unknown in more than 40% of patients [9]. We aimed to assess the predictive performance of a simplified PASO Score – the PASO Score without TOD – within a large multicenter cohort of patients who have undergone unilateral adrenalectomy between 2010 and 2016.

**Table 1:** The PASO Score as proposed by Burrello et al. [10].

Variable	Category	Points
Duration of Hypertension (months)	<120	7.5
	120–239	3.5
	>/ = 239	0
Sex	F	3
	M	0
BMI (Kg/m^2^)	<24	1.5
	24–29.9	0.5
	>/ = 30	0
Antihypertensive medication (DDD)	<3	6
	3–8.99	3
	>/ = 9	0
Target Organ Damage (left ventricle hypertrophy and/or micro-albuminuria)	Yes	0
	No	3
Nodule Size at Imaging (diameter, mm)	<13	0
	13–19	2
	>/ = 20	4

## 2 Materials and methods

This retrospective cohort study is reported according to the STROCSS 2019 guidelines [14].

### 2.1 Patients and data collection

For this validation study, patient data were used from the International CONNsortium Study Group database (NCT04761354, [15]), which has been previously extensively described [11,12,16,17]. In brief, patients with APA who were treated with unilateral adrenalectomy between 2010 and 2016 were included from 16 expert medical centers in North America, Europe and Australia (Supplement 1). The cohort represents the care delivered in daily clinical practice within the participating medical centers and, therefore, no strict inclusion or exclusion criteria were used regarding the workup to surgery (i.e., screening, case confirmation and subtype testing) [17]. In general, the elevated aldosterone-to-renin ratio (ARR) was used biochemically to indicate PA and computerized tomography (CT) and/or magnetic resonance imaging (MRI) and/or adrenal venous sampling (AVS) were used to determine disease laterality prior to surgery. Patients with missing values for preoperative or follow-up data regarding systolic blood pressure (SBP), diastolic blood pressure (DBP) or type and dosage of antihypertensive medication used were excluded (Supplement 1). Institutional review board approval was obtained in all participating centers.

### 2.2 Definitions and outcomes

Patients were evaluated and classified as complete, partial or absent clinical success using the PASO consensus criteria [9]. The patients with complete clinical success are those with postoperative normotension (i.e., SBP <140 mmHg and DBP <90 mmHg) during office blood pressure measurements without the aid of antihypertensive medication. Further details on the PASO consensus criteria on the outcomes are defined elsewhere [9]. The defined daily dose (DDD) is the average maintenance medication dose per day used to treat hypertension in adults and is determined using the World Health Organization Anatomical Therapeutic Chemical/DDD Index 2017 [18]. Biochemical data were reported as elevated or suppressed according to local cut-off values in each medical center. Hypokalemia was defined as potassium level below institutional reference range or when potassium supplementation was administered. CT and/or MRI scanning were used to measure the largest adrenal nodule size in millimeters. In cases when both CT and MRI were performed showing non-matching values, CT measurements were leading. In the PASO Score, TOD is defined as microalbuminuria and/or left ventricle hypertrophy. During the design of the International CONNsortium Study Group database, TOD was not included in the collection of the dataset. In the simplified PASO Score, we chose to assign TOD as absent to the whole cohort to eliminate the influence of the variable on the predictive performance. Although assigning TOD as present (0 points) or absent (3 points) to all patients will not cause a difference in AUC, we chose absent because the likelihood that TOD is absent is higher than present, as was shown within the original PASO cohort (63.4% absent) [10].

### 2.3 Statistical analysis

Continuous variables were presented as mean (±standard deviation) or median [interquartile range], depending on the distribution, and categorical variables were reported as counts (percentages). The dataset contained multiple missing values in variables used as predictor variables in the simplified PASO Score. These missing values were considered as missing at random and therefore imputed using the iterative Markov chain Monte Carlo method creating 20 datasets [19]. The simplified PASO Score was calculated and compared to the observed complete clinical success in each patient. The pooled AUC with subsequent 95% Confidence Interval (CI) was calculated by analyzing the simplified PASO score as a continuous variable. The pooled sensitivity, specificity, positive predictive value (PPV), negative predictive value (NPV) and accuracy were calculated using the original >16 cut-off. Since all patients were assigned 3 points for absent TOD, we performed sensitivity analyses by calculating diagnostic accuracy for >17, >18 and > 19 cut-offs as well. For analyzing the prognostic value of the simplified PASO Score to differentiate complete and partial clinical success from absent clinical success the >10 cut-off was used [10]. Additionally, we performed subgroup analyses within patients with or without AVS during the preoperative workup and within patients from either North America or Europe. Due to the low number of patients, we chose not to perform a subgroup analysis within the patients from Australia. Statistical analyses were performed using IBM SPSS Statistics 25 (IBM Corp, Armonk, New York).

## 3 Results

Based on the earlier stated inclusion criteria, a total of 380 (73.9%) out of 514 patients were eligible for analysis. Geographic location of these patients was North America (n = 245; 65.5%), Europe (n = 102; 26.8%) and Australia (n = 33; 8.7%) (Supplement 1). Baseline characteristics and frequencies of the PASO score predictors are shown in Table 2. The duration of hypertension, BMI and nodule size at imaging were missing in 12.1%, 7.9% and 15.5% of the patients, respectively. The other predictors and the PASO consensus criteria outcomes were known in all patients. The study group consisted of 165 (43.4%) females, mean age of 50.1 ± 11.3 years and mean BMI of 29.6 ± 5.8 kg/m^2^. The median duration of hypertension was 96 [36–144] months. The median DDD was 3.7 [2.0–5.6] and median nodule size at imaging was 15 [[11], [12], [13], [14], [15], [16], [17], [18], [19], [20]] mm. In 7.9% of patients the nodule size was based on MRI, because no CT was performed. Two hundred forty-one (63.4%) patients had AVS performed during their diagnostic workup. In all other patients, the laterality was based on CT or MRI. One hundred twelve (29.5%) patients had complete clinical success after surgery, whereas 212 (55.8%) and 56 (14.7%) patients had partial or absent clinical success, respectively.

**Table 2:** Baseline clinical characteristics of the study cohort (n = 380).

Variables	Number (%) or mean ± SD
Female	165 (43.4)
BMI (kg/m2) (n = 350)	29.6 ± 5.8
BMI (kg/m^2^)^b^	
<24	58 (15.3)
24–29	154 (40.5)
≥30	168 (44.2)
Duration of hypertension (months) (n = 321)^a^	96 [36–144]
Duration of hypertension (months)^b^	
<120	204 (53.7)
120–239	134 (35.3)
≥239	42 (11.0)
Defined daily dose^a^	3.7 [2.0–5.6]
Defined daily dose	
<3	149 (49.2%)
3–8	201 (52.9%)
≥9	30 (7.9%)
Tumor size at imaging (mm) (n = 334)^a^	15 [[11], [12], [13], [14], [15], [16], [17], [18], [19], [20]]
Tumor size at imaging (mm)^b^	
<13	140 (36.8%)
13–19	135 (35.6%)
≥19	105 (27.6%)
Age at surgery (years)	50.1 ± 11.3
Preoperative mean SBP (mm Hg)	149.9 ± 19.2
Preoperative mean DBP (mm Hg)	89.8 ± 12.7
ARR indicating PA (n = 309)	292 (94.5)
Elevated aldosterone level (n = 353)	193 (54.7)
Suppressed renin level/activity (n = 318)	214 (67.3)
Hypokalemia (n = 374)	275 (73.5)
Elevated creatinine level (n = 345)	60 (17.4)
AVS performed	241 (63.4)
Clinical success based on PASO consensus	
Complete	112 (29.5)
Partial	212 (55.8)
Absent	56 (14.7)

Abbreviations: SD = standard deviation; BMI = Body Mass Index; NA = Not Applicable; SBP = Systolic Blood Pressure; DBP = Diastolic Blood Pressure; AVS = Adrenal Venous Sampling; PASO = Primary Aldosteronism Surgical Outcome; IQR = Inter Quartile Range.

a Values not normally distributed given as medians [IQR].

b Including imputed data.

### 3.1 The simplified PASO score

**Fig. 1:**
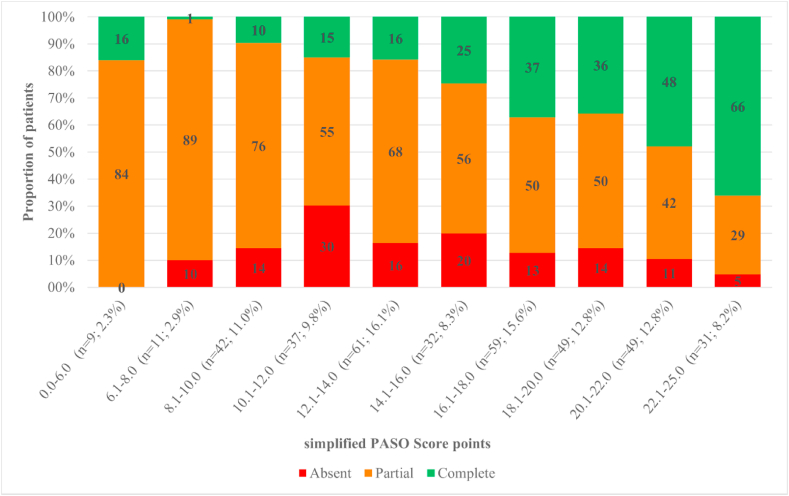
Stratification of clinical outcomes after unilateral adrenalectomy by simplified PASO Score. Legend: The histogram indicates the performance of the PASO predictor on the total cohort (n = 380) and shows the proportion of patients (y – axis, %) in each clinical outcome category (complete, green; partial, orange; absent, red) stratified by the simplified PASO Score (x – axis) in our cohort. Since all patients were assigned three points for absence of TOD, the minimum score for all patients is three. (For interpretation of the references to colour in this figure legend, the reader is referred to the Web version of this article.)

The most and least frequently observed simplified PASO Scores were 12.1–14.0 (16.1%) and 0.0–6.0 (2.7%), respectively (Fig. 1). The simplified PASO Score showed an AUC of 0.730 (95% CI 0.674–0.785) for predicting complete clinical success. For the original >16 cut-off, the sensitivity and specificity were 74.5% and 61.0%, respectively. A PPV of 44.4% and a NPV of 85.1% were calculated. The overall accuracy of the validation model was 65.0% (Table 3). As shown in Fig. 1, a simplified PASO Score >16 resulted in complete clinical success in at least 35.7% of patients which increased to 66.1% for the highest PASO Score of 22.1–25.0. Diagnostic accuracy measures for different cut-off values are reported in Table 3. In brief, higher cut-off values yielded a decreasing sensitivity and NPV, whereas specificity, PPV and accuracy increased.

**Table 3:** Predictive performance of the simplified PASO Score.

	Sensitivity	Specificity	PPV	NPV	Accuracy	AUC (95% CI)
Complete cohort (n = 380)	74.5%	61.0%	44.4%	85.1%	65.0%	0.730 (0.674–0.785)
Threshold 17	61.2%	70.4%	46.3%	81.3%	67.7%	0.730 (0.674–0.785)
Threshold 18	54.7%	74.9%	47.7%	79.8%	68.9%	0.730 (0.674–0.785)
Threshold 19	48.0%	82.1%	52.8%	77.3%	72.1%	0.730 (0.674–0.785)
AVS performed (n = 241)	68.0%	66.7%	45.5%	83.6%	67.1%	0.746 (0.674–0.817)
AVS not performed (n = 138)	84.9%	50.9%	42.2%	88.9%	61.0%	0.679 (0.586–0.772)
North America (n = 245)	76.5%	60.5%	41.6%	87.5%	64.8%	0.746 (0.679–0.814)
Europe (n = 102)	72.9%	63.0%	46.2%	84.0%	66.0%	0.705 (0.594–0.816)

The predictive performance of the simplified PASO Score was calculated by using complete clinical success as the outcome. Subgroup analyses were performed within the group of patients that did or did not undergo AVS preoperatively and within the North American and European populations. Similar to the study of Burrello et al. the AUC was calculated by analyzing the simplified PASO score as a continuous variable and the sensitivity, specificity, PPV, NPV and accuracy were calculated by using the >16 cut-off, except for the specified threshold calculations.Abbreviations: TOD = Target Organ Damage; AVS = Adrenal Venous Sampling; PPV = Positive Predictive Value; NPV = Negative Predictive Value; AUC = Area Under the Curve; CI = Confidence Interval.

A subgroup analysis in the 241 patients with preoperative AVS showed an AUC of 0.746 (95% CI 0.674–0.817). The PPV and NPV were 45.5% and 83.6%, respectively. Furthermore, a sensitivity and specificity of respectively 68.0% and 66.7% were observed, whereas the model accuracy was 67.1%. Within the subgroup of patients without AVS, the AUC was 0.679 (95% CI 0.586–0.772), PPV 42.2%, NPV 88.9%, sensitivity 84.9% and specificity 50.9%. The accuracy of the model in the subgroup without AVS was 61.0%. The AUCs within the subgroups of patients from North America and Europe were 0.746 (95% CI 0.679–0.814) and 0.705 (95% CI 0.594–0.816), respectively. Further comparison between patients from North America and Europe presented sensitivity and specificity of 76.5% versus 72.9%, 60.5% versus 63.0%, respectively. The PPV and NPV were 41.6% versus 46.2%, and 87.5% versus 84.0%, respectively. The accuracies were 64.8% versus 66.0%, respectively.

Evaluation of the prognostic value of the simplified PASO Score to predict complete and partial clinical success versus absent clinical success showed an AUC of 0.581 (95% CI 0.508–0.653) and a 83.2% sensitivity, 12.9% specificity, 84.6% PPV and 11.7% NPV were observed. The accuracy of the model was 68.5%.

## 4 Discussion

The PASO Score can be used to improve preoperative patient counseling which is necessary because complete cure of hypertension after surgery is no certainty. Since TOD status is not always readily available in general practice, we analyzed the predictive performance of a simplified PASO Score – i.e., the PASO Score without TOD – with the goal to increase the applicability of the model in daily practice. The results of our large multicenter cohort study showed an AUC of 0.730, which was lower compared to the original model within another large international cohort as presented by Burrello et al. (AUC 0.839) [10]. Yet, this prognostic performance can still be considered as moderate to good, because the model is used for patient counseling instead of deciding whether a patient should or should not undergo surgery. In addition, prediction models are known to ‘overfit’ in the original data and, therefore, show lower prognostic performance within external validation datasets [[20], [21], [22]]. Moreover, within the simplified PASO score we eliminated the prognostic value of TOD, which probably affects the AUC negatively. Based on this study, we believe that it is reasonable for clinicians to use the simplified PASO Score in daily clinical practice, when TOD status is unknown.

The proportion of patients with complete clinical success after the operation in our real-life cohort was lower (29.5%) compared to the 39.5% presented by Burrello et al. [10]. This lower clinical success rate may be attributed to the higher baseline BMI and DDD within our cohort compared to the PASO cohort, 29.6 versus 26.9 kg/m2 and 3.7 versus 2.5, respectively. Notably, our cohort was operated between 2010 and 2016 compared to between 1995 and 2015 in the PASO cohort. Therefore, the worldwide increase in primary hypertension over the last decades could also have influenced the lower rates of complete clinical success within our study [23]. Since our cohort represents daily clinical practice, the preoperative workup within our cohort was less stringent in following current guidelines compared to the preselected PASO cohort [17]. This is especially seen in the difference in the routine performance of AVS, 100% in the PASO cohort versus 63.4% in our cohort. Also, the follow-up duration was frequently shorter within our cohort. Although we did not find a clear difference in clinical outcomes between patients with shorter and longer follow-up in our previous studies within this cohort, this still could be of influence [11,12,16,17]. On the other hand, the PASO cohort, as described by Williams et al., only included patients with confirmed PA diagnosis according to the US Endocrine Society Guideline or the Japan Endocrine Society Guideline, and confirmed unilaterality by AVS [8,9,24]. Thereby, patients were excluded for the PASO Score study when data was missing for one of the PASO Score variables (duration of hypertension, sex, BMI, DDD, TOD or largest adrenal nodule at imaging). This resulted in the exclusion of 325 patients (46.1%) of the potentially eligible 705 patients. Subsequently, the remaining cohort was a subgroup of the original PASO cohort representing only 8 of the original 12 centers, which may have resulted in bias [25]. This resulted in a slightly higher rate of complete success compared to their original study, 39.5% versus 36.7%, respectively [9,10]. Additionally, in the original PASO cohort albuminuria was measured in 416 (59.0%) patients and left ventricle hypertrophy was measured in 455 (64.5%) patients [9]. So, missing data on TOD played a role in exclusion of patients. Also, this shows that TOD indeed is not readily available in all patients which questions the generalizability of the PASO Score and underscores the importance of validation of this simplified PASO Score.

The subgroup analysis of patients who underwent AVS during their preoperative clinical workup showed a slightly better predictive performance with an AUC of 0.746 (95% CI 0.674–0.817) than our complete cohort (AUC 0.730 (95% CI 0.674–0.785)). Subsequently, the subgroup analysis of patients who did not undergo AVS showed a slightly worse predictive performance with an AUC of 0.679 (95% CI 0.586–0.772), however, confidence intervals overlap between these two analyses. It can be hypothesized that the (simplified) PASO Score performs better in patients with AVS preoperatively. The high sensitivity (84.9%) of the simplified PASO Score in the non-AVS subgroup might indicate that patients in this group have a more favorable PASO Score profile, resulting in higher scores, compared to the AVS subgroup. So, this seems to imply that patients in our cohort are more likely to undergo AVS when they have an unfavorable preoperative profile (e.g., male, high BMI, high DDD, longer duration of hypertension, larger nodules). Also, we showed that the simplified PASO Score potentially performs slightly better in the population from North America than from Europe. In all subgroup analyses relatively wide confidence intervals were observed limiting direct comparisons between these groups. Similar to Burrello et al., we showed that the simplified PASO Score is not suited (AUC of 0.581) for differentiating between complete and partial versus absent clinical success and therefore the model cannot be used to predict patients who will not benefit from surgery [10]. It is thought that not achieving complete clinical success may be due to the presence of primary hypertension as an underlying comorbidity in patients with PA, leading to partial or absent clinical success after adrenalectomy. Since some of the prognostic variables for clinical success (BMI, duration of hypertension, DDD, TOD and sex) are also associated with primary hypertension, the (simplified) PASO Score most likely has great difficulty in separating and might not be able to predict partial and absent clinical success in patients with pre-existing primary hypertension.

In 2008 Zarnegar et al. developed the ARS which was the first simple and easy-to-use prediction score to predict resolution of hypertension after adrenalectomy for APA [13]. The ARS is based on four clinically and routinely available variables: ≤ 2 number of antihypertensive medications, ≤ 25 kg/m^2^ BMI, ≤ 6 years duration of hypertension and female sex. The ARS performed well within the development dataset of 100 patients with an AUC of 0.913. We recently showed, however, a lower predictive performance of the ARS with an AUC of 0.751 in the same International CONNsortium Study Group database as used in this study [16]. Thus, the simplified PASO Score and ARS had similar predictive performances within our cohort. Nevertheless, the PASO Score might be superior when TOD status is known, since adding information associated with the outcome (in this case TOD) will likely improve the AUC and thus the predictive performance of the prediction model. However, adding more information, i.e. variables, to a model comes at the cost of usability and might lead to overfitting.

Our study has several strengths and weaknesses. First, the major strength of this study is the large multicenter real-life cohort representing patients from three continents. Since we chose to not include or exclude patients based on the preoperative workup strategies, we believe our results to be representative of current daily clinical practice within the participating medical centers and most likely for others centers worldwide as well. Second, the PASO investigators included TOD in their prediction score as an extra presurgical variable and described it as an essential component of the evaluation of patients with PA. However, we doubt whether TOD status is analyzed in general practice. This also seemed to be the case within the original PASO cohort, since the TOD status was described as unknown in more than 40% of patients [9]. For that reason, incomplete data may limit the use of PASO Score in general practice. Therefore, we believe that the simplified PASO score is more representative for daily clinical practice due to exclusion of TOD. In this way, the results of this study can directly be utilized in daily care and will increase the worldwide applicability of the PASO Score. Third, although our cohort represents care delivered in daily clinical practice, the missing presurgical values regarding duration of hypertension, BMI and nodule size at imaging are a limitation of our study. However, the percentages of missing values were relatively low and because imputation of missing values is considered to be superior to complete case analysis we are confident that we minimized the effects of these missing values [19,26]. Fourth, the retrospective design of our study is another weakness. Fifth, our cohort did not have the data on long-term follow-up (>1 year), therefore we were not able to assess the predictive accuracy of the simplified PASO Score on the long-term. For example, Aronova et al. showed accurate prediction of the ARS (AUC 0.84) of the likelihood of complete clinical success beyond 1 year [27]. On the other hand, we recently showed that the effect of surgery on blood pressure is mostly seen in the first months and tends to remain stable over the long-term postoperative period, making a long-term predictive accuracy assessment unnecessary [28]. Finally, in our cohort not all patients underwent AVS preoperatively and a substantial proportion of patients had a relatively short follow-up. However, as mentioned earlier, our worldwide cohort represents current day-to-day clinical practice regarding the management of PA [11,12,16,17]. Therefore, difference in our cohort in presurgical workup made the results more applicable to daily clinical practice and for this reason heterogeneity in workup is an acceptable limitation. Additionally, we previously showed no differences in clinical success rates in our cohort in patients who did or did not underwent AVS preoperatively [12].

Ultimately the goal of prediction models is to inform patients and to improve preoperative counseling of patients regarding the chance of complete clinical success after unilateral adrenalectomy. As aforementioned, this is of importance since complete clinical success is no certainty [9,11,12]. In Fig. 1 there is a clear difference in percentages of complete clinical success in the groups with a simplified PASO Score higher than 16. Thus, one might use higher (simplified) PASO Scores (>16) in shared decision making as justification to perform a unilateral adrenalectomy, since these patients are likely to have a higher chance of complete clinical success postoperatively. Simplified PASO Scores lower than 16 show less chance of complete clinical success, although partial clinical success percentages remain high (>50%). Nevertheless, in the case of lower (simplified) PASO Scores, we believe that during shared decision making, the surgeon should make his/her decision to perform surgery based on individual patient analysis and the surgeon's expertise.

## 5 Conclusions

In conclusion, the present study shows that a simplified PASO Score without taking TOD into account is a useful tool for predicting outcome after adrenalectomy for PA in our worldwide cohort [10]. The simplified PASO score can be used in clinical practice when data on TOD are not readily available. In these cases, we advise to score TOD as absent and to apply three points in the PASO Score. Therefore, we believe these results should encourage the use of PASO Score in daily clinical practice.

## Ethical approval

Institutional review board approval was obtained for this retrospective cohort study in all participating centers.

## Sources of funding

This research did not receive any specific grant from funding agencies in the public, commercial, or not-for-profit sectors.

## Author contributions

Diederik Suurd: Methodology, Validation, Formal Analysis, Investigation, Data Curation, Writing – Original Draft, Visualization, Project Administration. Wouter Visscher: Methodology, Validation, Formal Analysis, Investigation, Data Curation, Writing – Original Draft, Visualization, Project Administration. Wessel Vorselaars: Conceptualization, Formal Analysis, Writing – Review & Editing, Supervision. Dirk-Jan van Beek: Conceptualization, Formal Analysis, Writing – Review & Editing, Supervision. Wilko Spiering: Conceptualization, Writing – Review & Editing. Inne Borel-Rinkes: Conceptualization, Writing – Review & Editing. Gerlof Valk: Conceptualization, Writing – Review & Editing, Supervision. Menno Vriens: Conceptualization, Writing – Review & Editing, Supervision. All authors gave final approval for the final version of the manuscript.

## Research registration number

1. Name of the registry: ClinicalTrials.gov.
2. Unique Identifying number or registration ID: NCT04761354.
3. Hyperlink to your specific registration (must be publicly accessible and will be checked): https://clinicaltrials.gov/show/NCT04761354.

## Guarantor

Prof. dr. Menno R. Vriens, corresponding author.

Prof. dr. Gerlof D. Valk, supervising author.

Diederik P.D. Suurd, MSc, first author.

Wouter P. Visscher, MD, first author.

## Consent

Since this study is based on a retrospective cohort/database, the ethical review committee has relieved us of the obligation to provide written informed consent for each included patient.

## Provenance and peer review

Not commissioned, externally peer-reviewed.

## Declaration of competing interest

The authors have nothing to disclose.
