# Supplementary material for: A simplified primary aldosteronism surgical outcome score is a useful prediction model when target organ damage is unknown – Retrospective cohort study
Source: Ann Med Surg (Lond). 2021 Apr 20;65:102333. doi: 10.1016/j.amsu.2021.102333 (PMC8091869; doi:10.1016/j.amsu.2021.102333)
Supplement: Multimedia component 1 [file mmc1.docx]

**
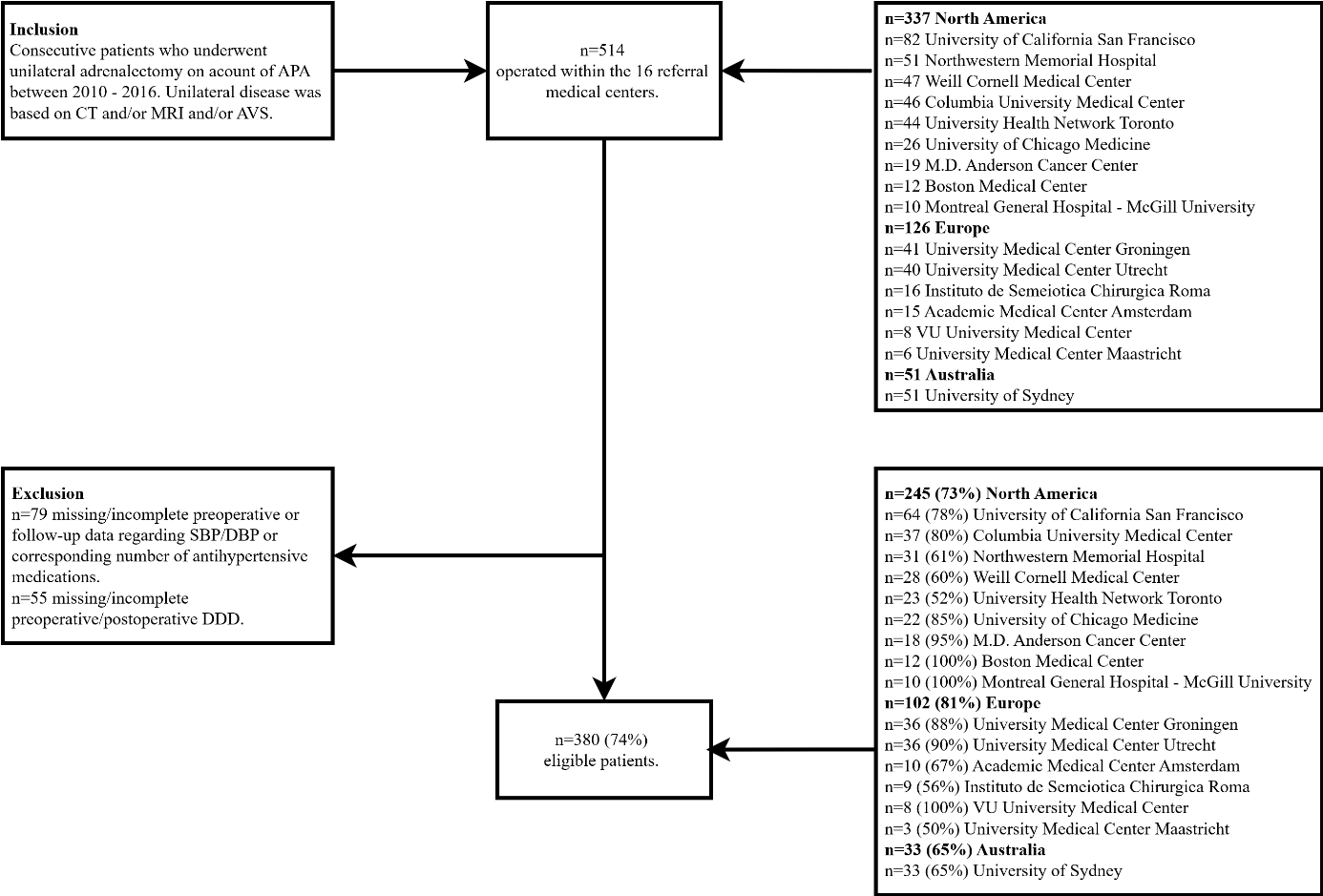
**

**Supplement 1.** Flowchart of the included patients.
Abbreviations: APA = Aldosterone Producing Adenoma; CT = Computed Tomography; MRI = Magnetic Resonance Imaging; AVS = Adrenal Venous Sampling; SBP = Systolic Blood Pressure; DBP = Diastolic Blood Pressure.
